# Including the Excluded in Antenatal Care: A Systematic Review of Concerns for D/deaf Pregnant Women

**DOI:** 10.3390/bs11050067

**Published:** 2021-05-01

**Authors:** Olufemi Timothy Adigun, Olugbenga Akinrinoye, Helen Ngozichukwuka Obilor

**Affiliations:** 1 Department of Educational Psychology and Special Education, Faculty of Education, University of Zululand, KwaDlangezwa, Durban 3886, South Africa; 2 Department of Paediatrics, College of Medicine, University College Hospital, Ibadan 200221, Nigeria; akinrinoye@gmail.com; 3 Department of Nursing, Faculty of Clinical Sciences, University of Ibadan, Ibadan 200221, Nigeria; 15hno@queensu.ca

**Keywords:** D/deaf, pregnancy, antenatal care

## Abstract

This paper presents global evidence derived from a systematic review of the literature on the issues of D/deaf pregnant women and antenatal care. A comprehensive search through four bibliographic databases identified a dataset of 10,375 academic papers, from which six papers met the inclusion criteria for in-depth analysis related to D/deaf pregnant women’s use of antenatal care/clinics. Findings from the analysis revealed four major concerns for D/deaf pregnant women who attended antenatal clinics for care. These concerns were communication difficulties, satisfaction with antenatal care services, attendance at antenatal clinics, and associated health outcomes. Based on the identified issues and concerns, it is recommended that pre- and in-service healthcare workers should be trained on how to communicate through sign language with their D/deaf patients. In addition, there is a need to rapidly expand the body of knowledge on the issues concerning antenatal care for D/deaf pregnant women vis-à-vis their relationship with healthcare workers in antenatal facilities.

## 1. Introduction

Globally, with no exception, every nation has yearly records of maternal and/or child mortality. Therefore, eradicating or reducing the phenomenon is a major concern for all stakeholders. While the problem remains a challenge, a committee of nations, through various international and national organizations/instruments, are striving to prevent maternal and/or child mortality. In the last three decades, despite all efforts, an estimate of about 810 maternal mortalities is recorded daily [1], while about 5.2 million infant deaths were estimated by the United Nations Children’s Fund [2] to have occurred in 2019, and about 3 million of these infant deaths occurred in sub-Saharan Africa.

With particular reference to Nigeria, child and maternal mortality issues remain a serious concern as the cases reported in the country constitute about 20% of the globally reported cases. In other words, Nigeria, with about 200 million inhabitants [3], experienced continually elevated infant mortality rates of about 87 per 1000 in 1990 to 100 per 1000 in 2003, while maternal mortality during the aforementioned period was estimated to be about 800 per 100,000 live births [1]. According to the WHO [1], between 600,000 and 900,000 near-misses of maternal mortalities occurred between 2005 and 2015 in Nigeria. It is noteworthy that although statistics on child and/or maternal mortality are carefully presented by UNICEF and the WHO, the percentage of the population of vulnerable women in the overall statistics, particularly those who are D/deaf and/or hard of hearing, is still unclear.

D/deafness is a condition that arises from the partial or total loss of the sense of hearing. In other words, variations in degrees of loss of the sense of hearing (moderate: 40–60 dB; severe: 61–90 dB; and profound: >90 dB) may further reinforce the dynamics and its heterogeneous nature. Globally, the population of D/deaf persons is yet to be ascertained, but about 5.3% of the world’s population is estimated to be living with hearing loss, with the majority living in low- and middle-income countries [4,5]. In sub-Saharan Africa, a 2012 report by the WHO indicates that about 7.4% of the adult population have hearing loss. Regarding the approximately 200 million population in Nigeria [3], Adigun and Iheme [6] remark that there has yet to be accurate data on the total population of the D/deaf, but [7] has reported that the percentage of the population of persons with hearing loss in Nigeria is about 23.7%. In an earlier study, Mba [8] states that one in every 1000th Nigerian is living with hearing loss. In other words, Mba’s projection implies that about 80 million or more Nigerians may be living with deafness [8].

Individuals who are D/deaf may be insensitive to sounds and thus lack the ability to actively respond to auditory-verbal stimuli [9,10]. The condition may arise at any stage of life. Thus, it is somewhat difficult to compare the emotional and psychological challenges associated with the acquisition of spoken language and its use in the context of prelingual deafness. Difficulties associated with coping with the loss of the ability to further use spoken language by individuals with postlingual deafness may further aggravate withdrawal tendencies and elevate psychosocial disorders [9,11]. Irrespective of their experiences in relation to deafness, it is pertinent to acknowledge the diversity in terms of identity and/or physiological conditions through the use of “D” and “d”. According to Pizzo [12] and Woodward [13], the “d” is often used to refer to the audiological condition and is mostly applied to individuals who use spoken languages, while “D” is often used to refer to those who are culturally bound by deafness. Deaf individuals believe primarily in the use of sign language as a means of communication. Despite the diversity in the use of “D” and ”d”, most often they are used interchangeably to denote persons with hearing loss. Therefore, this study jointly uses the capital and small letters (D/deaf) to denote individuals from the cultural-linguistic community.

The D/deaf are members of a minority group faced with communication challenges, especially where verbal communication is needed. Thus, the inability to actively communicate and interact adequately in situations where oral communication is needed has put the D/deaf at a disadvantage. According to Adigun [9], the communication gap between hearing members of the community and individuals who are D/deaf influences the level of marginalization and stigmatization experienced by the latter. Among the challenging issues related to D/deaf women’s health, sexual and menstrual health, as well as pregnancy and its care, remain a contemporary phenomenon. In particular, pregnancy care for D/deaf pregnant women vis-à-vis antenatal registration, visits, and associated care is still not clear. In addition, research evidence, especially in sub-Saharan Africa on the quality of antenatal care for D/deaf pregnant women and their interaction with health care workers in antenatal clinics, is limited.

Based on the foregoing, it is pertinent to ponder why the statistics available on maternal and child mortality rates as presented by the WHO and UNICEF at different times have yet to present data specific to vulnerable women, especially those who are D/deaf and/or hard of hearing. In addition, one may further query if D/deaf pregnant women’s health is a concern in the niche of public health practices.

Despite the unique characteristics of the population of D/deaf individuals, the structure and organization of the health care delivery system available to D/deaf individuals are yet to be adequately understood. Globally, there is limited research on the D/deaf’s mental and reproductive health status, especially among the female gender. More so, the interaction between the D/deaf and healthcare professionals is usually a challenge. Harmer [14] states that some of the difficulties in the patient–healthcare professional relationship stem from prejudice towards persons with special needs. Many health care practitioners may internalize bias towards patients who are D/deaf, thereby making it more difficult to establish appropriate communication strategies geared towards the provision of quality health care. Studies have indicated that communication difficulty with non-D/deaf peers is a major challenge faced by individuals who are D/deaf [14,15,16,17].

In addition, communication difficulties further hamper access to information on the available healthcare resources [14,17]. Ochieng, Atieli, Abongo, and Ouma [16] noted that D/deaf individuals were less knowledgeable in sexual/reproductive health knowledge and disease prevention compared with their hearing peers. Prejudice against persons with D/deafness is associated with poor access to reproductive health information [17]. Hence, as a result of communication difficulties and limited health information resources, the D/deaf, especially women, often access reproductive health information from informal sources such as peers within the Deaf community and/or family members [17,18,19]. Due to the informal health information, evidence from the literature shows that people with disabilities, especially women with deafness, experience worse health outcomes and are more likely to have unmet reproductive health care and antenatal care needs.

Over the last two decades, few studies have examined the barriers faced by women with various forms of disability when accessing health care services, especially on issues of access to and use of sexual and reproductive health care services [17,20,21,22,23]. Notably, women with disabilities are constantly faced with inequalities in receiving preventive health services and are less satisfied with the services received from health care providers [17,20]. Factors that prevent women with disabilities from accessing needed health care resources are the location of the health care facilities, long travel distance to health care facilities, and financial and structural barriers [21,22,23]. In addition, women with disabilities, including those with deafness, are faced with negative attitudinal challenges from health care workers. However, Arulogun, Titiloye, and Desmenu [21] state that the perceived negative attitudinal characteristics amongst healthcare workers towards patients with disabilities are perhaps due to occupational stress, lack of required training in dealing with persons with special needs, and/or communication difficulties.

Regrettably, lack of adequate education, knowledge and communication about health-related phenomena may heighten the risk of associated medical conditions and pregnancy complications among women who are D/deaf [15,16,21]. Mitra, Akobirshoev, McKee, and Iezzoni [24] have found that D/deaf women in the US are more likely to experience pregnancy complications, and have pre-term births and infants with low birth weights. Although health care policies have provided templates for the care of pregnant women and mandated health care workers to attend to the health needs of women attending the antenatal care/clinics, unfortunately, their practice and impact on deaf pregnant women are presently unclear. As indicated by Adigun and Mngomezulu [20], antenatal care is the medical care and support provided to pregnant women throughout pregnancy to promote the health of the mother and the babies.

The objectives of antenatal care/clinics are to offer regular check-ups by doctors and midwives through examinations and screenings for all pregnancy-related symptoms in order to facilitate health and psychosocial well-being, as well as to prevent potential health and pregnancy complications [25]. Therefore, early booking for antenatal care/clinics have been recommended by the WHO [25], and Kaswa, Rupesinghe, and Longo-Mbenza [26] have recommended them in the first trimester to achieve a stress-free gestational period.

Remarkably, a plethora of past studies have established variations in pregnancy- and antenatal-related issues among women without loss of the sense of hearing and/or speech [27,28,29]. Only a handful of research evidence is available among deaf pregnant women about antenatal care. Based on the foregoing, this study was designed to assess and collate global research evidence on the factors that influence antenatal care services to D/deaf pregnant women attending antenatal clinics. In addition, this study drew up a model for policies and practices for physicians, nurses, midwives, and other health care workers in antenatal clinics. Hence, this study was guided by the research question: what global evidence is available regarding the concerns for D/deaf pregnant women towards adequate inclusion and quality experiences in the antenatal care environment? To answer this question, we conducted a systematic review to identify and analyze the research on concerns related to D/deaf pregnant women and the antenatal care services received by them. Our findings will inform recommendations for future research, disability-inclusive health policy, and changes in practices of healthcare workers towards D/deaf pregnant women.

## 2. Materials and Methods

### 2.1. Inclusion and Exclusion Criteria

**Figure 1:**
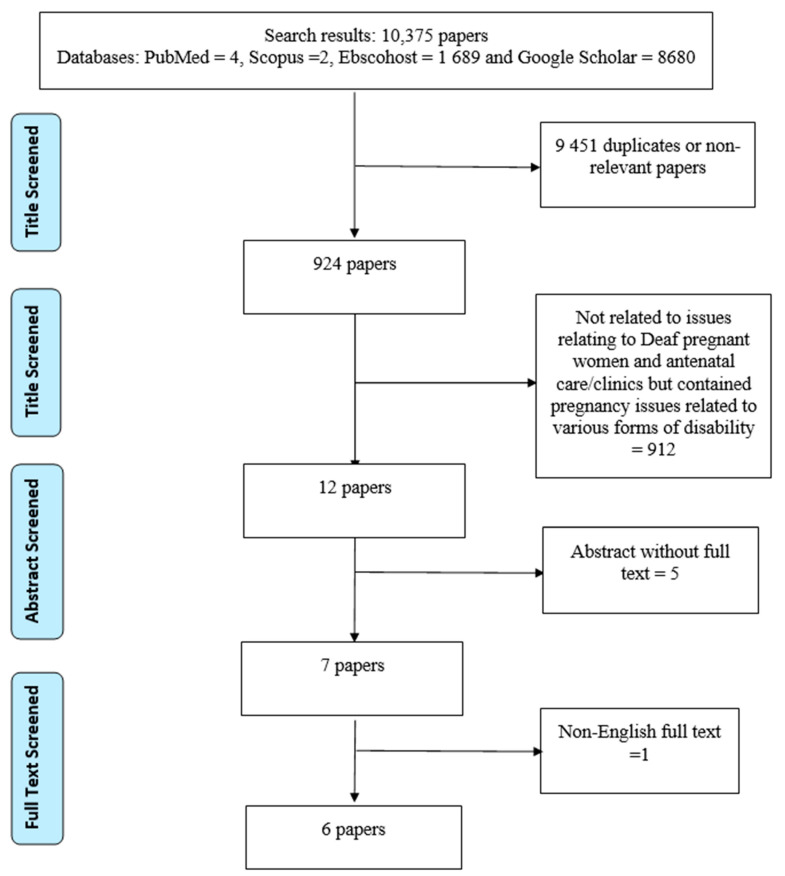
Flowchart of the systematic literature search.

Screening of the relevant studies was conducted by the author, based on specific inclusion and exclusion criteria. The author employed the assistance of two colleagues to review the selected studies using the approaches shown in Figure 1, using the criteria stated for the inclusion and exclusion of past studies, which were set before the literature search commenced. The inclusion criteria were as follows:

I. Studies on D/deaf pregnant women;
II. Studies published between the years 2000 and 2020;
III. Studies published in the English language;
IV. Studies that collected data from the D/deaf on issues of antenatal care; and
V. Full text articles.

The exclusion criteria were:

I. Studies published before 2000 and after 2020;
II. Abstracts, editorial comments, letters to the editor, and review articles;
III. Studies on postnatal care or other reproductive health issues;
IV. Non-English-language articles on D/deaf pregnant women and antenatal care; and
V. Studies on antenatal issues that incorporated pregnant women with other forms of disability.

### 2.2. Search Strategy

The Preferred Reporting Items for Systematic Reviews and Meta-Analyses (PRISMA) as recommended by Moher et al. [30] was adopted for this study. Relevant studies were identified through a systematic search in four bibliographic databases (PubMed, Scopus, Ebscohost, and Google Scholar). Articles in which “D/deaf pregnant women”, “pregnant women with hearing impairment”, plus “antenatal clinic/care” reflected in their titles were identified. Figure 1 shows the search strategy used in the study based on the above-stated inclusion and exclusion criteria. To further identify articles that met the inclusion criteria, a manual search of the reference lists of the relevant articles, theses, and dissertations was also conducted. The literature search was conducted between 28 January and 7 February 2021.

### 2.3. Selection of Studies

In accordance with the objective of this study, which sought to identify the global evidence available of the factors that prevented adequate inclusion of D/deaf pregnant women in antenatal care/clinics, open access/full text research papers of studies with participants were examined. That is, research studies that employed either a quantitative design, a qualitative design, a mixed methods approach, case reports, or a retrospective cohort study design were eligible for inclusion. In other words, opinion/theoretical/review papers and abstracts of closed access papers were excluded from the study. In this study, only articles on deaf pregnant women and antenatal care services were included. The literature reviewed in this study was limited to articles published in academic journals between 2000 and 2020. Only articles published in the English language were selected for the review, through the process shown in Figure 1.

### 2.4. Data Extraction

An eight-column form was designed and used to extract the data for this review from the identified articles. The form included information on the author(s), the year of publication, the title of the paper, the country of origin, the aim of the study, the study design, the participants, and the study findings. To ensure the validity of the information gathered, the authors sought the assistance of two colleagues: one from the Department of Educational Psychology and Special Education of a university in South Africa; and the other, a pediatrician in a tertiary health institution in Nigeria. The two colleagues assessed and critiqued the data extracted from all of the studies identified.

## 3. Results

### 3.1. Search Outcome

Based on the search for “D/deaf pregnant women and antenatal care” using various bibliographic databases (PubMed, Scopus, Ebscohost, and Google Scholar), a total of 10,375 records of published articles were found (See Figure 1). The records were screened in accordance with the inclusion and exclusion criteria set prior to the search. Out of the 10,375 articles, 9451 papers were removed because of duplication or non-relevancy of their contents to the aims of this study. Among the remaining 924 papers’ titles, a further 912 papers were considered unsuitable for the study, primarily because they were not related to issues relating to D/deaf pregnant women and antenatal care/clinics, but instead contained pregnancy issues related to various other forms of disability. Among the remaining 12 articles, only five article titles had freely accessible abstracts, while one paper with a relevant title was a non-English-language full-text paper. Finally, only six papers with full text met the inclusion criteria for the study. Table 1 presents the summary of the findings from the included studies. Out of the six included papers for this review, three were conducted among D/deaf women in the United States of America and one each in Nigeria, Saudi Arabia, and South Africa.

### 3.2. Study Design

As shown in Table 1, out of the three studies available that assessed issues of antenatal care among D/deaf pregnant women in the United States of America, two studies [31,32] used a retrospective cohort study approach, while the other [33] employed a quantitative research approach for data collection. The three other studies included and shown in Table 1 employed a mixed method research approach [34], a qualitative research approach [20], and a case report [35], respectively.

### 3.3. Participants

Overall, a total of 2105 D/deaf pregnant women participated in the included studies. Among all, the retrospective studies [31,32] had more D/deaf patients who attended antenatal clinics. Mitra et al. [31] included 1385 in their study, while a total of 645 D/deaf women were included in the article by Schiff, Doody, Crane, and Mueller [32]. Forty-two D/deaf pregnant women participated in the study by [34], while only nine D/deaf pregnant women were interviewed in the study by Adigun and Mngomezulu [20]. While Mustafa and Addar [35] reported on the case of a D/deaf pregnant woman in Saudi Arabia, O’Hearn [33] did a comparative study among 23 D/deaf pregnant women and 32 hearing pregnant women who were attending antenatal clinics in the USA.

### 3.4. Cumulative Main Findings

Findings derived from the included studies have been sub-divided into four sections. These are (i) communication, (ii) satisfaction with antenatal care/clinic services, (iii) attendance at antenatal care/clinics, and (iv) associated health outcomes.

**Communication:** Communication between the D/deaf pregnant woman, physicians, midwives/nurses, and other allied healthcare workers, could be difficult and demanding [35]. Mustafa and Addar [35] remarked in their case report that healthcare workers had difficulties in presenting medical issues to D/deaf pregnant patients through the use of pen and paper. According to the duo, communication with the D/deaf patient was time consuming, and thus it required a lot of patience to attend to such a patient. Similarly, in the comparative study conducted by O’Hearn [33], unlike hearing pregnant women, D/deaf pregnant women’s expectations were not met at the antenatal clinics because of a lack of sign language interpreters. Thus D/deaf pregnant patients relied on writing, using pen and paper for communication at every antenatal visit. Although the findings of Gichane, Heap, Fontes, and London [34] among 42 D/deaf pregnant women in the US were similar to what was observed by O’Hearn [33], Gichane, Heap, Fontes, and London [34] reported that there were limited sign language interpretation services at the hospitals visited by the study participants. More recent studies by Adigun and Mngomezulu [20] from Nigeria and Mitra et al. [31] from the United States of America expressed the communication difficulties experienced by D/deaf pregnant women who were attending antenatal clinics. According to the study by Adigun and Mngomezulu [20], communication challenges were one of the major determinants of the use of antenatal services by D/deaf women.

**Satisfaction with antenatal care/clinic services:** Satisfaction with healthcare services were associated with factors including accessibility, communication, and location. O’Hearn [33] reported that D/deaf pregnant women who attended antenatal clinics were less satisfied with the services rendered by physicians, midwives/nurses, and other allied health care workers when compared with the hearing pregnant women who attended the same clinics for antenatal care. Similarly, Adigun and Mngomezulu [20] in their study among nine D/deaf pregnant women from southwest Nigeria, found that D/deaf would-be-mothers attending antenatal clinics were not satisfied with the services rendered and received.

**Attendance at antenatal clinics:** Among the six studies included, the only case of a D/deaf pregnant woman presented by Mustafa and Addar [35] had five uneventful antenatal visits. Unlike the uneventful antenatal visits report by Mustafa and Addar [35] in their study, O’Hearn [33] reported that D/deaf pregnant women reported a lesser frequency of antenatal visits when compared to hearing pregnant women. Gichane et al. [34] stated that almost all participants in their study reported to the antenatal clinic at least once before their expected date of delivery. The study by Gichane et al. [34] did not indicate the gestational age at which the participants presented themselves for antenatal care, but almost all of the D/deaf pregnant women interviewed by Adigun and Mngomezulu [20] presented themselves for antenatal care during their second trimester.

**Associated health outcomes:** While the study of Mustafa and Addar [35] appreciated the clinical and psychological dexterities of the consulting physician, the study expressed concerns for D/deaf pregnant women who presented themselves for antenatal care and delivery when there was no active communication process. Although O’Hearn [33] did not indicate health risks among the participants of his study, the study raised concerns regarding the mental health of D/deaf pregnant women who presented themselves for antenatal care/service. According to O’Hearn [33], D/deaf pregnant women, when compared with their hearing counterparts, were less satisfied with the services received at every antenatal visit. Gichane et al. [34] also found that D/deaf pregnant women were more likely to be hospitalized for some days after delivery, and had a modestly increased risk of undergoing cesarean delivery. Similarly, an increased risk of pregnancy complications, chronic medical conditions, and adverse birth outcomes among D/deaf pregnant women was reported in the very recent study by Mitra et al. [31].

## 4. Discussion

The aim of this study was to assess and collate global research evidence on the concerns for D/deaf pregnant women regarding adequate inclusion and quality experiences in the antenatal care environment. In this study, the author elucidated issues that were capable of contributing to the exclusion of D/deaf pregnant women from quality antenatal care services. The authors found only six studies [20,31,32,33,34] that assessed antenatal issues with a specific focus on D/deaf pregnant women as participants through a systematic search of four bibliographic four databases (PubMed, Scopus, Ebscohost, and Google Scholar).

Findings from the reviewed studies established that D/deaf pregnant women experienced some forms of communication challenges when accessing antenatal care and services. Irrespective of gender, individuals with deafness experience communication difficulties mostly when the two-way communication involves healthcare professionals and the verbal exchange of ideas or thoughts [14,17,21,36]. In other words, where interaction and social relationships involved oral communication, individuals who were D/deaf seemed to be excluded. The exclusion of the D/deaf in communication processes that involved the verbal exchange of ideas or thoughts in social and or health discourses heightened the traumatic experiences associated with deafness [9,15,34,37]. Mustafa and Addar [35] indicated the use of pen and paper for communication in the case of an educated 30-year-old Deaf pregnant Saudi woman. Although Mustafa and Addar [35] acknowledged the difficulties associated with using pen and paper during this patient’s antenatal visit, one could at this point wonder how difficult it would have been if the patient was uneducated or did not understand the English language.

Meador and Zazove [15] averred that non–English-speaking Deaf were at the greatest risk for physician–patient miscommunication. D/deaf people, particularly those with prelingual deafness, had problems with language acquisition and development [37,38]. When compared to non-D/deaf individuals, persons who were D/deaf would not necessarily understand some health-related terms/words such as “allergic”, “constipation”, or even “nausea” [15,21,39,40]. The foregoing further established that D/deaf pregnant women had limited knowledge and inadequate understanding of many discourses that could ensue during their antenatal visits. Thus, this suggests the need to engage the services of sign language interpreters when D/deaf pregnant women are in for antenatal visits.

Amongst the six studies that met the criteria for inclusion in this study, only the study by Gichane et al. [34] indicated that health care facilities visited by their participants with deafness had limited provisions for sign language interpretation services. Some Deaf pregnant women who participated in the study by Adigun and Mngomezulu [20] made personal provision for sign language interpreters and took these interpreters with them to their antenatal visits. According to Gichane et al. [34], however, the use of personal sign language interpreters was a threat to ethical and human rights issues, as well as the right of persons to confidentiality. This implies that D/deaf patients may lose personal information and privacy.

The overall inference from the findings by this current study was that health care facilities were yet to incorporate sign language interpretation services. This finding supported the earlier findings of Witte and Kuzel [41] in their study, which assessed health care experiences among elderly deaf patients, that Deaf adults believed that health care workers seemed professionally unprepared to accommodate the communication needs of D/deaf patients. Arulogun et al. [21], in their study among purposively selected health care providers in Ibadan, Nigeria, established that participants had no training on how to relate and interact with D/deaf patients when they visited the hospital for consultation or treatment.

This study further noted that satisfaction with antenatal care/services among D/deaf pregnant women was low compared to hearing pregnant women [33]. The factors associated with the observed D/deaf low satisfaction with antenatal care are related to their level of education, socioeconomic status, access to health care facilities, communication with health care professionals, and location of the ANC clinic [20,33,34]. Several past studies have expressed concern about the dissatisfaction of D/deaf patients [20,37,42,43]. While the studies of Adigun and Mngomezulu [20] and Equy et al. [37] appreciated the psychosocial implications of pregnancy, the two studies expressed concern about the dissatisfaction reported with the services rendered at antenatal clinics to D/deaf pregnant women. According to Iezzoni, O’Day, Killeen, and Harker [42], the level of satisfaction expressed by D/deaf women with the services at the antenatal clinics was dependent on the communication processes that occurred during their visits.

Based on the above, and the findings obtained from the six studies included in this study, this author avers that the level of satisfaction with the services rendered by health care workers may be associated with attendance for antenatal care by D/deaf pregnant women. D/deaf pregnant women infrequently reported for antenatal care [33,34,35], and Adigun and Mngomezulu [20] stated that D/deaf pregnant women only booked antenatal visits late in their pregnancies. This finding was not surprising because communication difficulties between physicians and D/deaf patients could hamper excellent antenatal service delivery [14,15,16].

Miscommunication between physicians and D/deaf patients, particularly when providing antenatal care and services, could heighten the risk of pregnancy complications, chronic medical conditions, and adverse birth outcomes among these patients [31]. As found in this review, D/deaf pregnant women were susceptible to pregnancy and/or delivery complications [31,32,33]. Miscommunication and the use of non-professional interpreters such as family members or friends could lead to misdiagnosis, medical/treatment errors, and poor clinical outcomes [44].

## 5. Implications and Recommendations for Research, Policy, and Practice in Health Care

Women’s experiences with pregnancy are not the same. Irrespective of disabilities or hearing acuity, women generally have different emotional and physical manifestations during pregnancy. Physiological, structural, and neuroendocrine changes due to pregnancy have profound psychological effects on expectant mothers [45,46]. Depending on the psychosocial support and quality of care received by pregnant women, some experience a degree of anxiety in pregnancy, especially during their visits to antenatal clinics [47,48].

While there is a plethora of research evidence on the issues of pregnancy and the roles played by various health care professionals during antenatal care of women with hearing impairment, we found scanty research studies among D/deaf pregnant women. Hence, it is imperative that more research be commissioned to explore and investigate the current issues in antenatal care, with a large sample size of D/deaf pregnant women as active participants. Therefore, future research endeavors should recruit large samples of D/deaf pregnant women and antenatal care workers to further understand the complexities of antenatal care and service provision and the experiences of D/deaf pregnant women, physicians, midwives, and other health care workers. A cross-sectional, longitudinal, or mixed-method study on the complexities of antenatal care/services to D/deaf pregnant women will be of high value to health care policy formulation.

Based on this review’s findings on communication, we recommend that the medical school curricula and policies guiding medical training and re-training activities for physicians, midwives, and other health care workers involved in antenatal care should train them on the characteristics and needs of patients with D/deafness and/or other types of disabilities. Medical school curricula and modules should train students to the communication modes of D/deaf patients and accommodate the training of health care workers in sign language. Introducing pre-service health care workers to the rudiments of sign language and usage during medical training could equip them with beginner–intermediate level knowledge/skills in the sign language. We anticipate that this skill level in sign language could sustain the two-way communication process between D/deaf patients and healthcare professionals. In addition, we recommend that from about five to ten percent of in-service health care workers at various antenatal clinics should be trained in sign language. It is believed that the ability of health care workers to communicate with D/deaf pregnant women will increase their satisfaction and attendance at ANC services. In addition, skill in sign language and its usage by health care professionals and physicians will further enhance adherence to medical practice ethics on confidentiality and privacy of patients. Health care facilities should employ professional sign language interpreters in order to ease communication challenges between deaf patients and health care workers. This may also aid the confidentiality of the information provided by the health care workers to deaf patients.

Furthermore, medical practitioners at antenatal clinics need to be patient and devote more time to listening to D/deaf pregnant women. We believe a positive change in health care professionals’ attitude in listening to D/deaf patients could build these patients’ confidence in health care workers and the health system, increase their satisfaction, and improve maternal and child health care outcomes.

## 6. Limitations

This systematic review brought to the fore the limited research evidence available on issues concerning antenatal care and D/deaf pregnant women. The study only included studies that considered and made use of D/deaf participants as keywords and subject headings. Unfortunately, only a few studies that utilized a systematic research process were found. Opinion/theoretical articles on antenatal care issues and D/deaf pregnant women were not considered for inclusion in this review. Few studies were found to have considered active engagement of D/deaf pregnant women attending antenatal clinic/care, thus generalization of their findings had yet to be achieved. No studies considered a longitudinal research approach to understanding the issues concerning D/deaf pregnant women at antenatal clinics. In addition, no studies used a quasi-experimental research method with a designated control group to analyze the concerns at antenatal clinics with respect to D/deaf pregnant women. As just four bibliographic databases were used to search for articles using the keywords, it is possible that other unused bibliographic databases had articles pertinent to this study. Hence further relevant articles that considered “D/deaf pregnant women”, “pregnant women with hearing impairment”, and “antenatal clinic/care” may have unintentionally been excluded for analysis in this study. Lastly, this study did not assess the quality of the included studies. Future systematic review studies may consider inclusion of quality of studies included in their systematic reviews.

## 7. Conclusions

This study offered insight into the issues and concerns of D/deaf pregnant women regarding their antenatal care. The study provided an understanding of the strengths and weaknesses of the past studies on D/deaf pregnant women attending antenatal clinics for care, as well as the opportunities for and threats to achieving gratifying and satisfying antenatal care experiences for D/deaf pregnant women. This review’s findings demonstrated that there is a need to rapidly expand the body of knowledge on the issues concerning antenatal care for D/deaf pregnant women. The analysis of the included studies revealed four concerns that are associated with antenatal care for D/deaf pregnant women: communication, satisfaction with antennal care/clinic services, attendance at antennal care/clinics, and associated health risks. The findings suggest the need for active research engagement and analysis of the complexities in antenatal care for D/deaf pregnant patients. Furthermore, there is a need for healthcare professionals to understand the complexities associated with behavior and communication diversities of D/deaf pregnant women. Hence, we recommend the training of healthcare professionals in sign language or the integration of professional interpreters when dealing with D/deaf pregnant women.

## Figures and Tables

**Table 1:** General characteristics of the studies on D/deaf pregnant women and antenatal care included in this study.

Author(s)	Year	Title of Paper	Country	Study Aim	Study Design	Participants	Main Findings
Mustafa and Addar [35]	2000	Obstetric handling of a deaf patient	Saudi Arabia	To examine the experiences of obstetric handling of a deaf pregnant patient antenatally, during labor, and postpartum.	A case report.	One deaf woman.	Uneventful antenatal visits.
							Communication with patient through pen and paper could be difficult and time-consuming, and required a lot of patience.
							The clinical, psychological, and human aspects of the management were gratifying.
O’Hearn [33]	2006	Deaf women’s experiences and satisfaction with prenatal care: a comparative study	USA	To investigate factors impacting deaf patients’ satisfaction with prenatal care and prenatal care disparities between deaf and hearing women.	Quantitative research design.	23 deaf and 32 hearing women.	Deaf women were less satisfied than hearing women with physician communication and less satisfied with their overall care.
							Deaf women’s expectations about the provision of interpreter services being met or exceeded were significantly associated with their overall satisfaction.
							Hearing women had more prenatal care appointments and reported receiving more information from their doctors.
Schiff, Doody, Crane and Mueller [32]	2017	Pregnancy outcomes among deaf women in Washington State, 1987–2012	USA	To evaluate the association between deafness among pregnant women and selected adverse pregnancy and neonatal outcomes.	A retrospective cohort study	645 deaf women with single live births	Deaf women were more likely to have a delivery hospitalization of four or more days.
							Deaf women had a modestly increased risk of cesarean delivery.
Gichane, Heap, Fontes and London [34]	2017	“They must understand we are people”: Pregnancy and maternity service use among signing Deaf women in Cape Town	South Africa	To describe and compare the pregnancy outcomes and maternity service use of a sample of signing Deaf women of child-bearing age in Cape Town to the population of the Western Cape of South Africa.	Mixed method design.	42 Deaf women.	Almost all participants attended at least one antenatal appointment during their pregnancies, and all deliveries occurred at a health facility.
							Participants primarily relied on writing to communicate during antenatal visits and labor/delivery.
							Limited sign language interpretation services.
							Mistreatment by hospital staff.
Adigun and Mngomezulu [20]	2020	‘They forget I’m Deaf’: exploring the experience and perception of Deaf Pregnant women attending antenatal clinics/care	Nigeria	To explore the experiences and satisfaction of pregnant deaf women with antenatal care in Nigeria.	Qualitative research design.	Nine deaf pregnant women.	Participants registered/booked for antenatal care in their second trimester.
							Communication difficulties during their ANC (antenatal care) visits.
							Distance and location of the clinics, finance/cost, and health care professionals’ attitudes towards Deaf pregnant women.
							Satisfaction with ANC services at privately owned health care facilities as compared with publicly owned health care facilities.
Mitra, McKee, Akobirshoev, Valentine, Ritter, Zhang, McKee, and Iezzoni [31]	2020	Pregnancy, birth, and infant outcomes among women who are Deaf or Hard of hearing	USA	To conduct a more rigorous study using population-based, longitudinal linked data to compare pregnancy complications, birth characteristics, and neonatal outcomes between deaf or hard of hearing and non-deaf or hard of hearing women.	A retrospective cohort study.	1385 women who were Deaf and or Hard of hearing.	The deaf or hard of hearing women had an increased risk of chronic medical conditions and pregnancy complications.
							Deliveries of deaf or hard of hearing women were significantly associated with adverse birth outcomes.

## Data Availability

Not applicable.
